# Mindfulness-Oriented Recovery Enhancement remediates anhedonia in chronic opioid use by enhancing neurophysiological responses during savoring of natural rewards

**DOI:** 10.1017/S0033291721003834

**Published:** 2023-04

**Authors:** Eric L. Garland, Spencer T. Fix, Justin P. Hudak, Edward M. Bernat, Yoshio Nakamura, Adam W. Hanley, Gary W. Donaldson, William R. Marchand, Brett Froeliger

**Affiliations:** 1 Center on Mindfulness and Integrative Health Intervention Development, University of Utah, Salt Lake City, UT, USA; 2 College of Social Work, University of Utah, Salt Lake City, UT, USA; 3 Veterans Health Care Administration VISN 19 Whole Health Flagship site located at the VA Salt Lake City Health Care System, Salt Lake City, UT, USA; 4 Department of Psychology, University of Maryland; 5 Department of Anesthesiology, Division of Pain Medicine, Pain Research Center, University of Utah School of Medicine; 6 Department of Psychiatry, University of Utah School of Medicine; 7 Department of Psychiatry and Psychology, University of Missouri

**Keywords:** Analgesia, hedonic dysregulation, meditation, opioid use disorder, reward processing, savoring

## Abstract

**Background:**

Neuropsychopharmacologic effects of long-term opioid therapy (LTOT) in the context of chronic pain may result in subjective anhedonia coupled with decreased attention to natural rewards. Yet, there are no known efficacious treatments for anhedonia and reward deficits associated with chronic opioid use. Mindfulness-Oriented Recovery Enhancement (MORE), a novel behavioral intervention combining training in mindfulness with savoring of natural rewards, may hold promise for treating anhedonia in LTOT.

**Methods:**

Veterans receiving LTOT (*N* = 63) for chronic pain were randomized to 8 weeks of MORE or a supportive group (SG) psychotherapy control. Before and after the 8-week treatment groups, we assessed the effects of MORE on the late positive potential (LPP) of the electroencephalogram and skin conductance level (SCL) during viewing and up-regulating responses (i.e. savoring) to natural reward cues. We then examined whether these neurophysiological effects were associated with reductions in subjective anhedonia by 4-month follow-up.

**Results:**

Patients treated with MORE demonstrated significantly increased LPP and SCL to natural reward cues and greater decreases in subjective anhedonia relative to those in the SG. The effect of MORE on reducing anhedonia was statistically mediated by increases in LPP response during savoring.

**Conclusions:**

MORE enhances motivated attention to natural reward cues among chronic pain patients on LTOT, as evidenced by increased electrocortical and sympathetic nervous system responses. Given neurophysiological evidence of clinical target engagement, MORE may be an efficacious treatment for anhedonia among chronic opioid users, people with chronic pain, and those at risk for opioid use disorder.

## Introduction

Patients with chronic non-cancer pain are commonly treated with long-term opioid therapy (LTOT; ⩾90 days of opioid use) (Chou et al., 2009) despite risks including opioid misuse and opioid use disorder (OUD) (Chou et al., 2015). Although many patients take opioids as prescribed by their physician, approximately 25% of individuals receiving LTOT for pain engage in opioid misusing behaviors (Vowles et al., 2015) such as unauthorized dose escalation or using opioids to alleviate dysphoria (Butler et al., 2007). Hence, the drive to alleviate dysphoria with opioids is a key risk factor in the development of opioid misuse and OUD. Concomitantly, *anhedonia*, a reduced capacity to pursue, experience, and/or learn from pleasure (Rømer Thomsen, Whybrow, & Kringelbach, 2015), is a core feature of chronic pain that is magnified by opioid misuse (Garland, Trøstheim, Eikemo, Ernst, & Leknes, 2020; Trøstheim et al., 2020)

Exogenous opioids interact with endogenous opioid and dopamine systems (Johnson & North, 1992; Spagnolo et al., 2019) involved in regulating pain and reward. Over time, chronic opioid use in the context of prolonged pain is thought to produce anhedonia and blunt reward processing by dysregulating dopaminergic and opioidergic mechanisms underpinning healthy hedonic functioning (Volkow & McLellan, 2016). High dose use and misuse of opioid analgesics is thereby theorized to exacerbate pain-related hedonic dysregulation by (a) producing neuroadaptations in corticostriatal reward systems and (b) by magnifying antireward processes instantiated by limbic systems (e.g. extended amygdala) that mediate the release of signaling molecules including corticotropin-releasing factor, dynorphin, and substance *p* (Garland, Froeliger, Zeidan, Partin, & Howard, 2013; Koob, 2020). The consequent allostatic shift in brain reward threshold is thought to lead to anhedonia and dysphoria, compelling opioid dose escalation as a means of obtaining hedonic equilibrium. Insofar as anhedonia is often coupled with decreased allocation of attention to positive stimuli and reward-predicting cues (Armstrong & Olatunji, 2012; Brailean, Koster, Hoorelbeke, & De Raedt, 2014; Huhn et al., 2021; Rømer Thomsen, 2015), opioid misusing chronic pain patients demonstrate reduced autonomic and attentional responses to natural reward cues (Garland, Froeliger, & Howard, 2015a, 2017c) relative to medication-adherent pain patients and healthy controls. Patients with OUD exhibit a similar attenuation of event-related potentials (ERP) of the electroencephalogram (EEG) during attention to natural reward cues, and this impairment predicts the risk of relapse (Lubman et al., 2009). Specifically, the late positive potential (LPP) of the EEG indexes motivated attention to emotionally salient stimuli, which elicit a larger LPP than neutral stimuli reaching maximum amplitude at parietal sites (Pz) between 400 and 2000 ms after image onset (Hajcak & Foti, 2020; Schupp et al., 2000). Beyond deficits in ‘bottom-up’ attention to natural reward, chronic pain patients who engage in high-dose opioid use and misuse also exhibit an inability to use ‘top-down’ cognitive control to up-regulate sympathetic responses to natural reward cues, as evidenced by blunted skin conductance levels (SCL) as early as several seconds after stimulus onset (Hudak et al., 2021). SCL has been shown to index attention to emotionally salient stimuli (Bradley, Codispoti, Cuthbert, & Lang, 2001) and to covary with the LPP and arousal ratings of images representing natural rewards (Cuthbert, Schupp, Bradley, Birbaumer, & Lang, 2000). Further, the anticipation of reward results in increases in SCL that can be modulated by emotion regulation strategies – with concomitant effects on corticostriatal activity (Delgado, Gillis, & Phelps, 2008; Smith, Rigney, & Delgado, 2016). Although SCL is a non-specific biomarker that can also index the orienting reflex (Sokolov, 1963) and conditioning responses (Oe et al., 2016), attenuated SCL observed when people on LTOT attempt to up-regulate their response to natural reward might be driven by dysfunctional corticostriatal mechanisms and indicative of impaired emotional attention and arousal. Given this interpretation, neurophysiological deficits during attention to natural reward cues may contribute to symptoms of anhedonia and deficient reward processing in people with chronic pain on LTOT.

To date, there are no known efficacious treatments for anhedonia and concomitant deficits in attention to natural reward associated with chronic opioid use (Kiluk, Yip, DeVito, Carroll, & Sofuoglu, 2019). Blunted reactivity to reward-predicting stimuli and receipt of non-drug rewards has been observed in the dorsolateral prefrontal cortex and ventral striatum among patients receiving medication for OUD (i.e. methadone) (Moningka et al., 2019). This finding suggests that anhedonia and reward processing remain untreated by one of the most effective first-line medications for OUD. Cognitive-behavioral therapy (CBT) often includes behavioral activation to increase engagement in rewarding life activities. However, being physically present with a natural reinforcer may not counter cognitive biases that divert attention away from natural reward. In that regard, a recent review found no evidence that behavioral activation (or CBT as a whole) significantly improves anhedonia in chronic opioid users (Kiluk et al., 2019). Specific attentional training in savoring may be needed to overcome attentional bias and increase sensory-perceptual contact with the rewarding stimulus (Garland, 2020).

*Savoring* involves attending to the pleasant features (e.g. visual, auditory, gustatory, olfactory, tactile, or kinesthetic) of a naturally rewarding stimulus and the resultant positive emotions and pleasurable sensations that emerge during contact with the stimulus (Bryant & Veroff, 2017). Mindfulness training is theorized to promote savoring of natural rewards by stabilizing and reorienting attention from distraction onto the pleasant stimulus, and then by deepening meta-awareness of positive emotional and interoceptive responses to the stimulus (Garland, 2020). In that regard, a novel cognitive intervention, Mindfulness-Oriented Recovery Enhancement (MORE) unites training in mindfulness and savoring skills to remediate reward dysregulation among chronic opioid users. MORE has been tested in multiple randomized controlled trials (RCTs) for chronic pain patients prescribed LTOT. Across these trials, MORE decreased chronic pain symptoms, opioid misuse, craving, and illicit drug use, while simultaneously increasing positive affect and self-reported savoring (Cooperman, Hanley, Kline, & Garland, 2021a; Garland et al., 2014b, 2019c, 2021). Additionally, pilot studies found MORE increased cardiac-autonomic and ERP markers of attention to natural reward cues (Garland, Froeliger, & Howard, 2014a, 2015b, 2019a). However, no study has assessed whether MORE's effects on modulating neurophysiological responses to natural reward cues are associated with improvements in anhedonia among people on LTOT.

To that end, we conducted an ancillary mechanistic sub-study overlaid on a clinical trial (NCT02935621) to test whether participation in MORE *v.* an active control condition [supportive group (SG) psychotherapy] would occasion increased LPP and SCL responses during attention to natural reward cues, and whether such increased neurophysiological responses would be associated with clinical improvements in anhedonia. We hypothesized that (1) MORE would be associated with significantly greater increases in ‘bottom-up’ neurophysiological responses to natural reward cues than the SG, (2) MORE would increase the capacity to up-regulate these neurophysiological responses during savoring, and (3) the effects of MORE on reducing subjective anhedonia would be associated with increases in neurophysiological response to natural reward cues.

## Materials and methods

### Participants

In this ancillary mechanistic study, we added a neurophysiological assessment protocol to an ongoing clinical trial (NCT02935621) where EEG outcomes were not proposed as part of the original clinical trial design. This mechanistic study evaluated neurophysiological (EEG and SCL) data from a sample of Veterans with chronic pain receiving LTOT (*N* = 63). Primary clinical outcomes from NCT02935621 will be reported elsewhere. Individuals who provided complete pre-post treatment EEG data (Table 1) were included in the present analysis (see online Supplementary material for CONSORT Diagram). Participants (83% male; mean age = 60.3 ± 10.1 years) were recruited from VA primary care and pain clinics, and met inclusion criteria if they had taken opioids for at least the past 90 days (mean opioid use duration = 9.8 ± 8.3 years) and reported chronic non-cancer pain (mean pain duration = 15.9 ± 13.9 years). The mean (±s.d.) opioid dose in morphine milligram equivalents was 71.7 ± 168.1 mg. On the Mini-International Neuropsychiatric Interview (MINI; Sheehan et al., 1998), 63% met the criteria for major depressive disorder (MDD), 16% for generalized anxiety disorder (GAD), and 11% for post-traumatic stress disorder (PTSD). Participants were excluded if they had previously engaged in a formal MBI or for active suicidality or psychosis as assessed by the MINI. Participants were financially compensated. The protocol was approved by the University of Utah IRB and VA Salt Lake City Health Care System Research and Development Committee, and all procedures complied with standards set forth in the Helsinki Declaration of 1975.

**Table 1.** Baseline demographic and clinical characteristics (*N* = 63) of patients with chronic pain on long-term opioid therapy (LTOT) treated with Mindfulness-Oriented Recovery Enhancement (MORE) or a supportive group (SG) psychotherapy control condition

Measure	MORE (*n* = 32)	SG (*n* = 31)	Between-groups test statistic *p* value
Female, *N* (%)	6 (19%)	5 (16%)	0.59
Age, *M* ± s.d.	60.2 ± 9.8	58.1 ± 10.3	0.43
Race, *N* (%)			0.47
White	25 (78%)	27 (87%)	
African American	2 (6%)	2 (6%)	
Hispanic/Latino	2 (6%)	0 (0%)	
Native American/American Indian	2 (6%)	0 (0%)	
Other	1 (3%)	2 (6%)	
Primary pain location, *N* (%)			0.52
Back	18 (55%)	21 (68%)	
Joints	7 (18%)	3 (11%)	
Neck/shoulders	3 (9%)	2 (22%)	
Extremities	1 (6%)	3 (7%)	
Other	3 (12%)	2 (4%)	
Opioid type^a^			0.32
Hydrocodone	13 (41%)	8 (26%)	
Oxycodone	9 (28%)	9 (29%)	
Tramadol	11 (34%)	11 (35%)	
Morphine	1 (3%)	5 (16%)	
Methadone	1 (3%)	1 (3%)	
Other	0 (0%)	1 (3%)	
Opioid use duration (years)	8.5 ± 6.5	11.1 ± 9.8	0.22
Average pain, *M* ± s.d.	5.2 ± 1.5	5.1 ± 1.8	0.89
Morphine equivalent daily dose, *M* ± s.d.	56.6 ± 127.0	87.8 ± 202.9	0.46
Opioid use disorder	13 (41%)	11 (36%)	0.67
Non-opioid substance use disorder	3 (9%)	2 (7%)	0.67
Major depressive disorder	20 (63%)	18 (58%)	0.46
Post-traumatic stress disorder	2 (6%)	5 (16%)	0.20
Generalized anxiety disorder	8 (25%)	2 (6%)	0.05
Anhedonia (SHAPS), *M* ± s.d.	24.3 ± 6.3	22.8 ± 5.6	0.32

a Subjects were allowed to enter more than one opioid type. SHAPS, Snaith Hamilton Anhedonia and Pleasure Scale.

### Procedures

Following screening, participants who gave written informed consent completed demographic and clinical assessments, including the Snaith Hamilton Anhedonia and Pleasure Scale (SHAPS) (Snaith et al., 1995). Then, they completed a laboratory-based, natural reward responsiveness task (see below) during which EEG and SCL were recorded. Participants were informed that they would be randomized to a behavioral treatment group that would help them to cope with pain, stress, and opioid-related problems by providing either mindfulness training or group support. After the pre-treatment assessment, participants were randomly allocated to MORE or an SG control using simple randomization in blocks of varying sizes (2–4) to preserve allocation unpredictability. After the 8-week MORE and SG treatments, participants returned to the laboratory to again complete the natural reward responsiveness task. The SHAPS was assessed again at post-treatment and 2- and 4-month follow-ups.

### Interventions

The manualized MORE intervention program provided training in mindfulness, reappraisal, and savoring skills as techniques to cope with opioid craving, pain, and negative affect. Group sessions were 2 h long and led by a psychologist. Sessions included training in mindful breathing and body scan techniques to help patients self-regulate pain and opioid craving, reappraisal training to decrease negative emotions, and savoring training to amplify natural reward processing. With respect to savoring, specifically, participants were trained to mindfully focus attention on the sensory features of a pleasant object presented in the session (e.g. a rose), and then to appreciate and enjoy any positive emotions or pleasurable body sensations arising during the encounter with the pleasant stimulus. Then, participants were asked to engage in daily 15 min home practice of mindfulness as well as practicing savoring with naturally-occurring pleasant objects and events in everyday life.

To control for non-specific factors including attention by a caring professional, therapeutic expectancy, and social support, we employed a manualized active SG control in this study. The SG consisted of 8 weekly, 2 h, process-oriented, Rogerian group psychotherapy sessions, in which a psychologist facilitated emotional expression and discussion of topics pertinent to chronic pain and opioid use/misuse. This client-centered SG format was validated in prior RCTs of MORE (Garland et al., 2014b, 2019c). SG participants were asked to engage at home in 15 min of journaling a day on chronic pain and opioid-related themes. To prevent treatment diffusion, participants in the SG condition were instructed to not engage in mindfulness training during the course of the study. A clinician with 15+ years of experience conducted clinical supervision and reviewed session recordings to monitor therapist adherence to the MORE and SG treatment manuals and maintain intervention fidelity.

### Assessment of natural reward responsiveness

In an experimental laboratory session, participants were presented with images representing natural rewards or neutral cues. On each trial, participants were first shown a fixation cross for 500 ms, followed by 250–500 ms jittered blank screen and then an image and instruction label for 5000 ms. Across 64 trials, cues were presented in a randomized, event-related design.

Participants were instructed to View or Regulate responses to natural reward stimuli. On View trials, participants were instructed to simply attend to images of naturally rewarding stimuli (e.g. social affiliation, natural beauty, athletic victories, etc.) validated in prior studies (Garland, Bryan, Nakamura, Froeliger, & Howard, 2017c) or neutral images (e.g. people with neutral facial expressions, household objects) whose basic visual properties were matched to the natural reward cues. On Regulate trials, to approximate mindful savoring techniques and conform with typical ‘increase positive’ instructions on emotion regulation tasks (Froeliger et al., 2017), participants were instructed to imagine experiencing the positive event occurring in the image, and to focus on and appreciate the pleasant aspects of the image and their own positive emotional response to the image. In a training session prior to psychophysiological assessment, participants practiced this regulatory strategy and described their experience to a trained research assistant to ensure comprehension of the instructions. Psychophysiological assessment did not commence until participants could accurately describe the implementation of regulatory instruction.

#### EEG

EEG was continuously recorded from 10 midline scalp sites (Fz, F3, F4, FC1, FC2, FCz, Cz, CP1, CP2, PZ) using an active sensor cap with Ag/AgCl electrodes (actiCap GmbH, Herrsching, Germany). All recordings were collected by an actiCHamp amplifier (Brain Products GmbH, Gilching, Germany). Data were acquired at a sampling rate of 500 Hz, a resolution of 0.489 *μ*V and an amplification cutoff of 140 Hz, with impedances kept below 10 kΩ.

EEG data preprocessing was conducted using a custom MATLAB (MATLAB version 9.3.0,.713579 (R2017b), 2017) script set developed by the authors, containing both original and EEGLAB (Delorme & Makeig, 2004) functions, while ERP analysis was performed in the Psychophysiological Toolbox (Bernat, Williams, & Gehring, 2005). A low-pass filter of 50 Hz was applied to the continuous data, and then ERP epochs were created beginning 1000 ms pre-stimulus and ending 2000 ms post-stimulus. For each individual, epochs were ranked according to the number of extreme (> ± 150 mV) data points across all channels, and the worst 5% of epochs were removed. Additionally, individual channels were interpolated across all data if they exceeded the threshold of 5 standard deviations in the domains of kurtosis and activity probability. After baseline (500–100 ms pre-stimulus) correction occurred, each epoch was evaluated separately and channels with extreme (> ± 150 mV) data points were interpolated only for that epoch, while epochs with more than two bad channels were rejected and removed from the data. A final visual inspection was conducted to remove epochs with unusual artifacts. ERP component scores were extracted and exported for further statistical analyses. For hypothesis testing, we assessed activity at Pz where the LPP was maximal, consistent with previous research (Foti & Hajcak, 2008; Schupp et al., 2000).

#### Skin conductance

Skin conductance sensors were placed on the middle phalanx of the index and middle fingers of the non-dominant hand. A BIOPAC MP160 amplifier provided a constant voltage (0.5 V) across the two electrodes. Change relative to a 500 ms pre-stimulus baseline was computed in half-second epochs.

#### Anhedonia

The SHAPS (Snaith et al., 1995) consists of 14 items tapping the pleasure experienced from a variety of natural rewards (e.g. being with family, a beautiful landscape, receiving praise), rated on a Likert-type scale (1 = *strongly agree*, 4 = *strongly disagree*). Although both 2- and 4-point SHAPS scoring formats have been reported (Trøstheim et al., 2020), because we and others have found the greater variability of the 4-point scoring format to be sensitive to effects of chronic pain and substance use (e.g. opioids) (Trøstheim et al., 2020), we selected the 4-point scoring format for this study. SHAPS total scores ranged from 14 to 56, with higher scores indicating higher levels of anhedonia.

#### Positive affect

The Positive and Negative Affect Schedule-short form (PANAS-SF) was used to assess positive affect with 10 items rated on a Likert-type scale (1 = *not at all*, 5 = *extremely*) (Watson, Clark, & Tellegen, 1988).

#### Reward experience

One item from the Applied Mindfulness Process Scale (AMPS; Li, Black, & Garland, 2016), ‘In the last week, I enjoyed the little things in life more fully’, was used as a process measure at pre- and post-treatment to assess the impact of MORE skills (*v.* the SG) on reward experience, rated on a Likert-type scale (0 = not at all, 4 = extremely).

### Statistical analysis

This mechanistic study was powered to detect the effects of MORE on neurophysiological processes. Power analysis conducted with G*power 3.1 indicated that 60 subjects were required to detect a statistically significant (*p* < 0.05, two-tailed), small-moderate effect size (*η*_partial_^2^ > 0.04) ‘within-between’ interaction via repeated-measures ANOVA; power = 0.80.

Given the canonical time window of the LPP and our *a priori* hypothesis, we used repeated-measures ANOVA (RM-ANOVA) to examine the effects of treatment on EEG response from 400 to 2000 ms to capture the maximal peak deflection of the LPP. Following convention (Hajcak & Foti, 2020; Schupp et al., 2000), we defined the LPP as average voltage in successive time windows: 300–600, 600–1000, 1000–1500, and 1500–2000 ms. In contrast, we examined the effects of treatment on SCL across the entire stimulus viewing period from 0 to 5000 ms in 500 ms windows, because we did not have a strong *a priori* hypothesis about the time window whereby MORE would exert its effect on sympathetic responses. Prior to hypothesis testing, we assessed for baseline equivalence in the pre-treatment data, and then adjusted the LPP and SCL for neutral stimulus processing by subtracting activation to neutral cues from activation to reward cues for each epoch. To test our first hypothesis that MORE would be associated with significantly greater increases in ‘bottom-up’ reward responsiveness than the SG over time, we assessed the interaction of Treatment (MORE *v.* SG) with Time (Pre- *v.* Post-treatment) on the physiological response to natural reward stimuli. To test our second hypothesis that MORE may amplify ‘top-down’ conscious up-regulation of reward responsiveness through savoring, we assessed the interaction of Treatment(MORE *v.* SG) with Strategy (View *v.* Regulate) and Time (Pre- *v.* Post-treatment) on the physiological response to natural reward stimuli. Both LPP and SCL analyses tested the main effect of time window, as well as its interaction with Treatment, Time, and Strategy, to determine if effects differed across the time-course of the physiological signal. Because MORE and SG differed at baseline in GAD baseline diagnosis, and because opioid dose could confound physiological responses, we controlled for these variables in sensitivity analyses. Significant (*p* < 0.05) main effects and interactions from RM-ANOVAs were investigated with Bonferroni-adjusted planned *post hoc* contrasts, with violations of sphericity addressed with Greenhouse Geiser-corrected df.

Next, to perform an intention-to-treat analysis of the effects of treatment on SHAPS scores over time, we used linear mixed modeling (LMM) in SPSS 27 with maximum likelihood estimation and fixed effects consisting of a time factor and between-subjects treatment factor (MORE *v.* SG). The parameter of interest was the Treatment × Time interaction, and the model was specified with a patient-level random intercept and a repeated covariance structure determined by AICC information criteria. One-sample *t* tests were used to compare anhedonia scores to meta-analytically derived reference values for healthy subjects. In exploratory post-hoc analyses, we used similar LMMs to examine treatment effects on PANAS and AMPS item scores.

Finally, we examined correlations between neurophysiological responses (LPP and SCL) and anhedonia scores in the follow-up period. In an exploratory post-hoc analysis, we also examined neurophysiological correlates of positive affect and reward experience. We then conducted a path analysis in the R version 4.0.3 Lavaan package using maximum likelihood estimation with robust (Huber–White) standard errors to evaluate neurophysiological responses as a mediator of treatment effects (MORE *v.* SG) on anhedonia.

## Results

### Baseline equivalence

There was no significant between-groups difference by Treatment condition on the LPP (*F*_1,61_ = 2.45, *p* = 0.12, *η*_partial_^2^ = 0.03) or SCL at baseline (*F*_1,51_ = 2.24, *p* = 0.14, *η*_partial_^2^ = 0.04), nor were the Treatment × Strategy or Treatment × Strategy × Window interactions significant, demonstrating baseline equivalence between groups.

### Treatment effects on LPP response

**Fig. 1.**
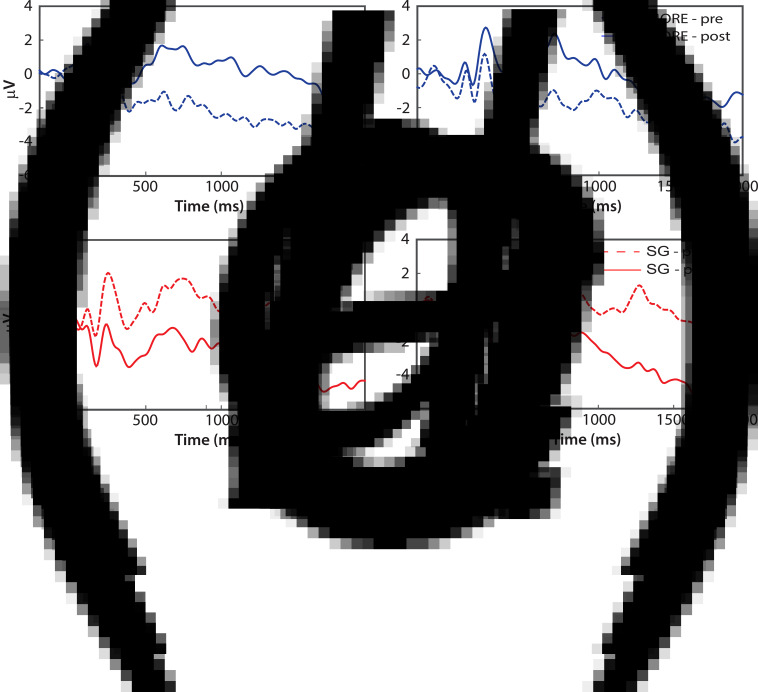
Changes in parietal (Pz) late positive potential (LPP) during (*a*) (top left) passive viewing of natural reward (i.e. View trials) from pre- to post-treatment with Mindfulness-Oriented Recovery Enhancement (MORE); (*b*) (top right) up-regulation of responding to natural reward (i.e. Regulate trials) from pre- to post-treatment with MORE; (*c*) (bottom left) passive viewing of natural reward (i.e. View trials) from pre- to post-treatment with supportive group (SG) psychotherapy; (d) (bottom right) up-regulation of responding to natural reward (i.e. Regulate trials) from pre- to post-treatment with SG.

**Fig. 2.**
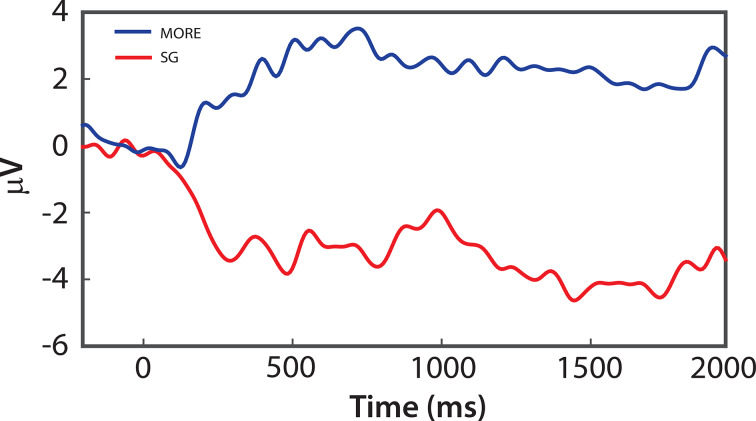
Difference waves (post-treatment minus pre-treatment) depicting effects of Mindfulness-Oriented Recovery Enhancement (MORE) *v.* supportive group (SG) psychotherapy on parietal (Pz) late positive potential (LPP) response to natural reward cues, averaged across View and Regulate trials.

Effects of treatment on the LPP (Fig. 1) were examined with RM-ANOVA. A significant Treatment × Time interaction was observed, *F*_1,61_ = 4.80, *p* = 0.03, *η*_partial_^2^ = 0.07, indicating that regardless of strategy, MORE was associated with significantly greater increases in LPP response to natural reward cues across the LPP than the SG, regardless of strategy (Fig. 2). However, neither the Treatment × Strategy × Time interaction (*F*_1,61_ = 0.006, *p* = 0.94, *η*_partial_^2^ < 0.001), nor the Treatment × Strategy × Time × Window interaction (*F*_3,183_ = 1.84, *p* = 0.16, *η*_partial_^2^ = 0.03), was significant, indicating that the effect of MORE (relative to the SG) did not significantly differ between regulatory strategies over time; relative to the SG, MORE increased LPP from pre- to post-treatment to a comparable degree for both the View and Regulate conditions across the entire LPP and did not differ by time window. Similarly, neither the main effect of Time, the main effect of Strategy, nor the main effect of Treatment was significant. In the sensitivity analysis, the Treatment × Time interaction remained significant, *F*_1,58_ = 5.41, *p* = 0.02, *η*_partial_^2^ = 0.09.

### Treatment effects on SCL response

**Fig. 3.**
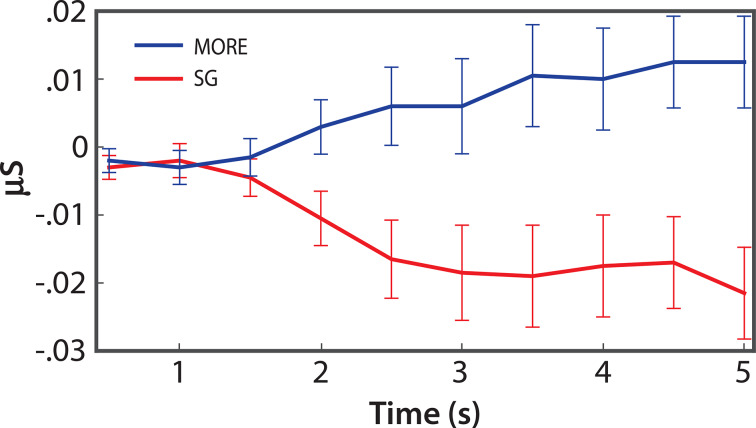
Difference waves (post-treatment minus pre-treatment) depicting effects of Mindfulness-Oriented Recovery Enhancement (MORE) *v.* supportive group (SG) psychotherapy on SCL response to natural reward cues, averaged across View and Regulate trials for 500 ms windows through the 5 s cue presentation.

Effects of treatment on SCL (Fig. 3) were examined with RM-ANOVA. Emerging from this analysis was a significant Treatment × Time × Window interaction, *F*_1.5,91.4_ = 4.54, *p* = 0.020, *η*_partial_^2^ = 0.07. Inspection of the data indicated that regardless of strategy, MORE was associated with significantly greater increases in SCL response to natural reward cues than the SG; simple contrasts indicated this treatment effect reached statistical significance around the 2000 ms time window, and peaked by 4500 ms (*F*_1,58_ = 7.49, *p* = 0.008, *η*_partial_^2^ = 0.11). However, none of the other higher-order interaction effects were significant, and neither the main effect of Time, the main effect of Strategy, nor the main effect of Treatment was significant. In the sensitivity analysis, the Treatment × Time × Window interaction remained significant, *F*_1.5,495_ = 4.28, *p* = 0.025, *η*_partial_^2^ = 0.07.

### Treatment effects on anhedonia, positive affect, and reward experience

In LMM of SHAPS scores, the Treatment × Time interaction was significant, *F*_(1,47.09)_ = 6.42, *p* = 0.015, indicating that participants in MORE exhibited a greater decrease (Δ SHAPS = −3.00 ± 1.14) in anhedonia through the 4-month follow-up than those in the SG (Δ SHAPS = 0.52 ± 1.16). At the pre-randomization assessment, SHAPS scores among participants (23.6 ± 0.8) significantly differed (*p* < 0.001) from the established SHAPS reference value for healthy individuals (20.2 ± 0.03). By 4-month follow-up, SHAPS scores among participants in MORE (21.3 ± 1.3) did not significantly differ (*p* = 0.41) from the established SHAPS healthy reference value, whereas SHAPS scores among SG participants (22.9 ± 1.1) remained significantly different from the SHAPS healthy reference value (*p* = 0.018).

With regard to PANAS positive affect, although the main effect of Time was significant *F*_(1,51.66)_ = 7.41, *p* < 0.001, the Treatment × Time interaction was not, *F*_(1,51.66)_ = 1.43, *p* = 0.25, indicating that participants in both MORE and those in supportive psychotherapy reported significant increases in positive affect over time. Finally, with regard to the AMPS reward experience item, the Treatment × Time interaction was significant, *F*_(1,57.57)_ = 4.13, *p* = 0.047, indicating that participants exhibited a greater increase in reward experience by post-treatment than the SG.

### Association between neurophysiological mechanisms and anhedonia, positive affect, and reward experience

**Fig. 4.**
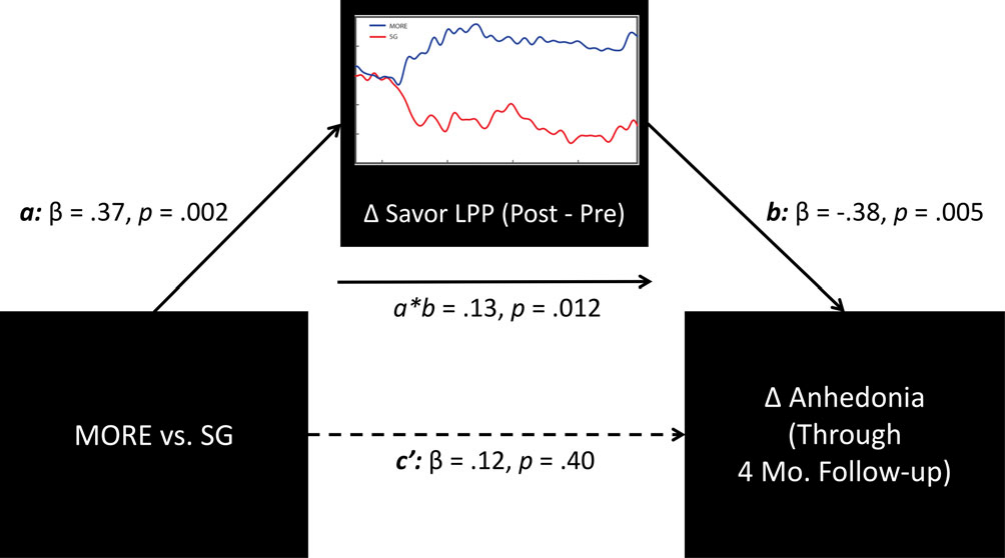
Path model indicating that the effect of Mindfulness-Oriented Recovery Enhancement (MORE) *v.* supportive group (SG) psychotherapy on reducing anhedonia by 4-month follow-up was statistically mediated by increasing parietal (Pz) late positive potential (LPP) to natural reward cues.

Across the entire sample, pre-post increases in LPP during the regulate strategy correlated with lower SHAPS scores throughout the follow-up, *r* = −0.34, *p* = 0.01. Controlling for opioid use and MDD, GAD, and PTSD diagnoses as potential confounders, the correlation between increases in the LPP during the regulate strategy and decreased anhedonia remained significant (*p*s ranged from 0.021 to 0.029). Given this association, we conducted a path analysis with the R Lavaan package (Fig. 4). The indirect effect (*a* × *b*) was significant (*p* = 0.012, 95% CI 0.30–2.35), demonstrating that increased LPP during the regulate strategy mediated the effect of MORE on reducing anhedonia. However, neither SCL nor the LPP during the view strategy was significantly associated with anhedonia. Although positive affect was not associated with SCL or LPP, the AMPS reward experience item at post-treatment was positively correlated with the LPP (1500–2000 ms window) during viewing of natural reward cues, *r* = 0.26, *p* = 0.047.

## Discussion

Here we obtained neurophysiological evidence in support of the hypothesis that MORE increases motivated attention to natural reward cues in chronic pain patients on LTOT – effects that were associated with decreased anhedonia. Relative to an active SG psychotherapy control condition, participants in the MORE group exhibited heightened LPP and SCL responses to natural reward stimuli. In individual difference analyses, increases in LPP activity during savoring predicted reductions in anhedonia symptoms through a 4-month follow-up.

Findings suggest that training in attention allocation to the positive exteroceptive and interoceptive features of a stimulus context (i.e. savoring) through MORE can amplify LPP markers of motivated attention to natural reward stimuli. MORE's effects were similar on View and Regulate trials; though MORE provides training in proactive up-regulation of reward via savoring, it seems to enhance motivated attention to natural reward cues in a bottom-up fashion even when no conscious attempts to savor are made. Hypothetically, repeated practice of savoring may restructure the reward value of non-drug rewards, such that over time, no proactive efforts to up-regulate reward are needed; indeed, automatic (implicit) emotion regulation has been observed and indexed by the LPP (Zhang & Zhou, 2014) – with top-down regulation of responding to emotional stimuli exerting long-lasting, dose-dependent effects on bottom-up neural reactivity to those stimuli (Denny, Inhoff, Zerubavel, Davachi, & Ochsner, 2015). These data replicate and extend prior findings of enhanced LPP responses to natural reward cues from smaller pilot studies of MORE (Garland, Froeliger, & Howard, 2015b, 2019a). Hypothetically, the increases in LPP response during attention to natural reward cues observed in the present study might be undergirded by corticostriatal activation. This speculation is consistent with neuroimaging data indicating that up-regulation of positive emotion is subserved by activation in medial PFC, caudate, and putamen (Li et al., 2018), and pilot work in smokers indicating that MORE enhances activity in these same brain regions during savoring (Froeliger et al., 2017).

SCL during attention to natural reward cues also increased following 8 weeks of treatment with MORE, with no difference between the View and Regulate conditions. In contrast, SCL and the LPP decreased among participants in the SG – likely a function of habituation. Skin conductance habituation has been reliably demonstrated during viewing of positive affective images (Codispoti, Ferrari, & Bradley, 2006). Rather than exhibiting the normative habituation response, participants in MORE exhibited heightened SCL to natural reward stimuli over time. SCL is a known index of the attentional orienting response and sympathetic arousal during threat and reward (Löw, Lang, Smith, & Bradley, 2008). The observed LPP and SCL effects suggest that MORE increased attention to natural reward stimuli and enhanced subsequent motivational arousal.

Further, based on a recent systematic review (Kiluk et al., 2019), these data provide the first evidence that a behavioral intervention of any kind can reduce anhedonia in people with chronic pain on LTOT. MORE significantly decreased anhedonia symptoms, effects that were associated with increased LPP during attention to natural reward cues during savoring, suggesting that this technique – integral to MORE – may be especially important for remediating reward processing deficits. In that regard, MORE also increased self-reported reward experience as measured by a mindfulness process measure. However, despite prior studies where MORE has shown efficacy for increasing positive affect (Garland et al., 2017b, 2019c), in the present study both MORE and supportive psychotherapy increased positive affect to a comparable degree. These findings suggest that MORE has unique effects on targeting anhedonia above and beyond the generalized improvements in emotional well-being produced by supportive psychotherapy. That said, increased SCL occasioned by MORE was not correlated with improved anhedonia, so converging sympathetic evidence was not obtained. In addition, multiple other factors could have confounded the observed association between increased LPP during savoring and decreased anhedonia, including changes in anxiety, depression, PTSD – psychological variables that have all been shown to be treated by MORE in prior studies (Cooperman et al., 2021a; Garland, Roberts-Lewis, Tronnier, Graves, & Kelley, 2016). Replication studies should examine associations between the neurophysiological effects of MORE and improvement of such factors to determine whether increased LPP during savoring is more robustly and specifically related to anhedonia than to other measures.

The current study had several other important limitations. First, because that the present analysis did not examine MORE's effects during attention to negative emotional stimuli, it is possible that MORE might increase attentional responses to emotionally salient stimuli of any valence. Yet, prior studies have indicated that MORE decreases attention to both negatively valenced stimuli (Garland et al., 2019b; Garland & Howard, 2013) and drug-related stimuli (Garland, Baker, & Howard, 2017a, 2019a), suggesting that the observed increases in neurophysiological response might be selective for positive stimuli (i.e. natural reward cues). The selectivity of this effect should be determined in future studies involving a range of stimulus valences. Furthermore, without a corresponding behavioral measure of reward such as self-reported liking or wanting, or a performance-based measure of motivation, like the EEFfrT (Treadway, Buckholtz, Schwartzman, Lambert, & Zald, 2009), the task used in the present study cannot assess whether MORE impacts reward responsiveness above and beyond attention to natural reward cues. Next, a high-density electrode montage would have allowed source localization for inferences about the brain networks involved. However, given the vulnerable nature of the study participants, we selected a limited set of electrodes to minimize participant burden. Also, because participants were instructed to take opioids as prescribed on the day of the experiment to prevent withdrawal-related cognitive and neurophysiological disturbances, the acute pharmacological effects of opioids may have influenced neurophysiological responses. That said, the observed effects of MORE on LPP responses remained significant in sensitivity analyses controlling for opioid dose. Future studies could examine mindfulness-induced modulation of EEG oscillations in opioid-naïve chronic pain patients and in patients following stabilized medical titration from LTOT. Finally, the study had a modest sample size comprised largely of older white male veterans, limiting the generalizability of study findings; future investigations should employ larger, more racially diverse samples to examine associations between neurophysiological responses during attention to natural rewards and clinical outcomes.

In summary, following 8 weeks of treatment with MORE, patients receiving LTOT for chronic pain exhibited increased electrocortical and sympathetic nervous system responses during attention to natural reward cues that predicted decreases in anhedonia 4 months after treatment ended. In light of neurophysiological evidence of clinical target engagement, adequately powered, full-scale clinical trials are now needed to test the efficacy of MORE as a means of treating anhedonia and reward deficits among chronic opioid users, people with chronic pain, and those at risk for OUD. Given that anhedonia is the hallmark of depression, and depression may exacerbate the risk for opioid misuse and OUD among chronic pain patients (Emery & Akil, 2020), future, large-scale trials should aim to replicate these effects in patients on LTOT with chronic pain and comorbid MDD.

## Supplementary material

For supplementary material accompanying this paper visit https://doi.org/10.1017/S0033291721003834. click here to view supplementary material
