# Supplementary figures and images for: Mindfulness-Oriented Recovery Enhancement remediates anhedonia in chronic opioid use by enhancing neurophysiological responses during savoring of natural rewards
Source: Psychol Med. 2021 Oct 14;53(5):2085–94. doi: 10.1017/S0033291721003834 (PMC10106294; doi:10.1017/S0033291721003834)

**Supplement**

Consort Flow Diagram


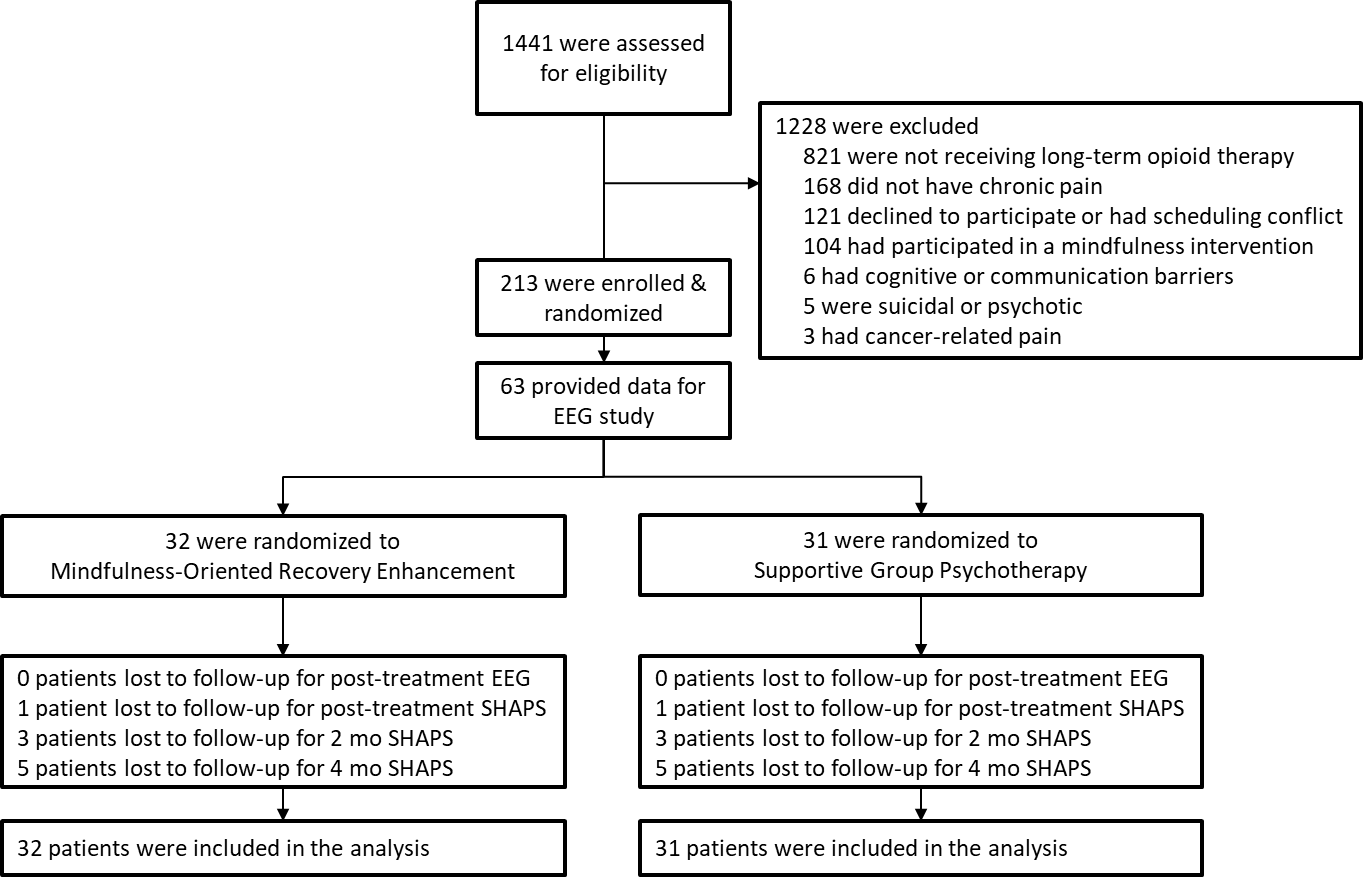

Supplement: Supplementary file 1 [file S0033291721003834sup001.docx]
